# Progranulin deficiency in the brain activates an insulin signaling pathway that may promote neurodegeneration

**DOI:** 10.1016/j.isci.2026.115720

**Published:** 2026-04-12

**Authors:** Mini P. Sajan, Geetika Aggarwal, Joel Jihwan Hwang, Denise M. Smith, Denise R. Cooper, Mildred Acevedo Duncan, Barbara C. Hansen, Andrew D. Nguyen, Robert V. Farese

**Affiliations:** 1 Division of Endocrinology and Metabolism, Department of Internal Medicine, University of South Florida Morsani College of Medicine, Tampa, FL 33602, USA; 2 Tampa Veterans Administration Medical Center, Tampa, FL 33612, USA; 3 Division of Geriatric Medicine, Department of Internal Medicine, Saint Louis University School of Medicine, St. Louis, MO 63104, USA; 4 Department of Pharmacology and Physiology, Saint Louis University School of Medicine, St. Louis, MO 63104, USA; 5 Institute for Translational Neuroscience, Saint Louis University, St. Louis, MO 63104, USA; 6 Department of Chemistry, University of South Florida School of Arts and Sciences, Tampa, FL 33620, USA

**Keywords:** genetics, molecular biology, neuroscience

## Abstract

Molecular mechanisms in frontotemporal dementia (FTD) and Alzheimer’s disease (AD) are obscure. FTD can result from loss-of-function progranulin mutations, although pathogenetic consequences are uncertain. Progranulin insufficiency also increases human AD risk, and progranulin treatment improves mouse AD. Furthermore, AD and FTD risks are abetted by obesity/diabetes-induced hyperinsulinemia and hyperactivation of brain insulin signaling, and progranulin deficiency activates insulin signaling in fat and liver. Here, we found progranulin deletion in mouse brain increased activation of IRS-1 and activities of downstream PKC-λ/ι, NF-κB and mTOR, but diminished IRS-2 and Akt. Similarly, in microglial cells, progranulin deletion increased, and progranulin treatment diminished, activation of IRS-1, PKC-λ/ι, NF-κB, and mTOR. These progranulin-related changes in IRS-1 activation were due to JNK-mediated phosphorylation of inhibitory serine-302/307 residues in IRS-1. Progranulin deficiency in brain selectively activates an IRS-1-dependent insulin signaling pathway, and the resultant increases in inflammation and impaired autophagy/lysosomal function may augment progranulin deficiency-related neuropathology.

## Highlights

• In brain, insulin acts through IRS-1 to activate PKC-λ/ι, NF-κB, and mTOR
• Progranulin acts via JNK to inhibit IRS-1 and signaling to PKC-λ/ι, NF-κB, and mTOR
• Progranulin deficiency activates IRS-1, thereby impacting inflammation and autophagy
• IRS-1 hyperactivity in brain may abet neurodegeneration via PKC-λ/ι, NF-κB, and mTOR

## Graphical abstract

**Figure:**
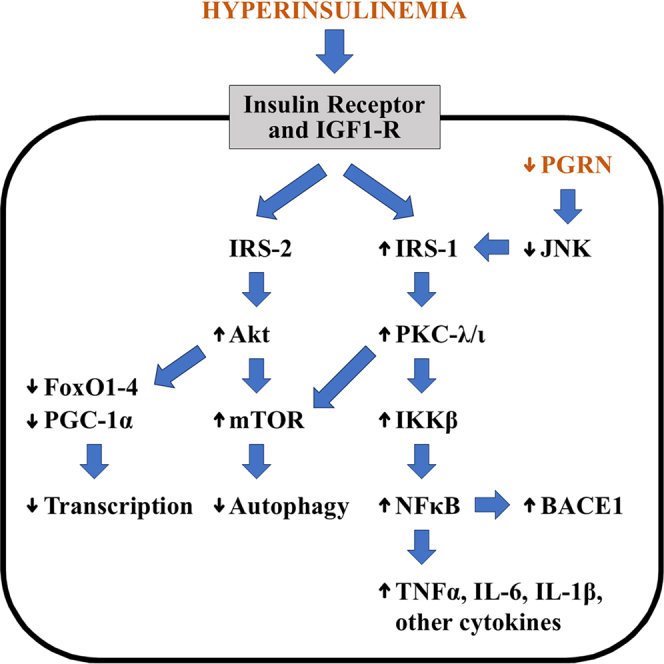

## Introduction

Hereditary frontotemporal dementia (FTD) and sporadic Alzheimer’s disease (AD) are increasingly prevalent, relentlessly progressive, devastating neurodegenerative disorders for which effective treatments have lagged as their underlying pathogenetic mechanisms have remained obscure. Pathologically, AD is characterized by overproduction of plaque-forming amyloid-beta (Aβ) peptides and hyperphosphorylation of tangle-forming tau proteins, particularly in hippocampal brain areas. FTD is not characterized by interneural Aβ-plaques and intraneuronal phospho-tau (p-tau) tangles (although they may occur to some extent)^1^ but often reflects heterozygous, loss-of-function mutations in the granulin (*GRN)* gene in chromosome 17 and subsequent haploinsufficiency of progranulin (PGRN) that leads to neurodegeneration, particularly in frontal and temporal cortices of the brain.^2^^,^^3^^,^^4^ Interestingly, *GRN* mutations and polymorphisms (including rs5848) have also been seen in some forms of AD,^5^^,^^6^^,^^7^^,^^8^^,^^9^ suggesting that reduced PGRN levels may increase AD risk.

The *GRN* gene encodes for PGRN, which is a lysosomal and secreted protein that contains 7.5 cysteine-rich granulin domains. The full-length PGRN proprotein can be proteolytically cleaved to yield individual granulins, which are the bioactive units.^10^^,^^11^^,^^12^ PGRN has pleiotropic and cell type-specific effects. In microglia and macrophages PGRN suppresses inflammation,^13^^,^^14^^,^^15^ and in neurons it promotes neurite outgrowth^16^^,^^17^ and mitochondrial function.^18^^,^^19^^,^^20^ Additionally, extracellular PGRN has neurotrophic and growth factor-like properties that involve activation of several cellular signaling pathways.^17^^,^^20^^,^^21^^,^^22^^,^^23^^,^^24^ These observations suggest that PGRN has some activities that are likely lysosome-independent.

Here, we examine the possibility that PGRN-deficient FTD and AD may share aberrations of insulin signaling that could be pathologically important in the brain. Despite the etiological and anatomical differences described previously, general inflammatory and neurodegenerative processes are common to FTD and AD. Importantly, multiple studies have found that risk of both AD^25^^,^^26^^,^^27^^,^^28^ and FTD^29^^,^^30^ are increased by the presence of both type 2 diabetes mellitus (T2DM) and its widely prevalent, frequent forerunner, the “metabolic syndrome” (MetS), which is commonly initiated by caloric excess that leads to insulin resistance in fat, liver and muscle, and thus to glucose intolerance, hyperinsulinemia, obesity, hyperlipidemia and hypertension (i.e., the MetS). Indeed, in a series at the Mayo Clinic, 80% of AD patients had either overt T2DM (half) or glucose intolerance (half).^25^ These risks presumably reflect hyperactivity of certain components of the insulin signaling system that is caused by hyperinsulinemia,^31^^,^^32^^,^^33^^,^^34^ owing to systemic insulin resistance that is present throughout the entire course of the MetS, which is commonly followed by development of hyperinsulinemic phases of T2DM. On the contrary, glucagon-like peptide-1 receptor agonists (GLP-1RAs; such as Ozempic), which act by inhibiting the brain appetite center causing weight loss and decreases in insulin resistance and hyperinsulinemia, have recently been found to be associated with reduced risk of developing AD in individuals with T2DM.^35^^,^^36^

As one mechanism for increasing AD risk in MetS/T2DM, hyperinsulinemia in mice and monkeys with diet-induced MetS/T2DM was found to strongly/maximally activate the brain insulin receptor and both Akt and atypical protein kinase C (aPKC), i.e., two major serine/threonine (Ser/Thr) protein kinases that operate downstream of insulin receptor substrate-1 (IRS-1) and/or insulin substrate-2 (IRS-2), which activate Akt and aPKC via phosphatidylinositol 3-kinase (PI3K)^32^^,^^33^^,^^34^ (in brain, PKC-λ/ι is the major/sole 70–75 kDa aPKC, and PKC-ζ is largely a 50 kDa, constitutively active kinase called PKMζ).^34^ In short, any resistance to insulin that may have existed in brains of these MetS/T2DM mice and monkeys was largely/fully overcome by activation of “spare receptors,” i.e., levels of insulin or insulin-like growth factor-1 (IGF-1) receptors (which are readily activated by elevated insulin levels)^37^ above those needed to maximally activate downstream processes that are largely controlled by Akt and PKC-λ/ι. And, whereas modest, transient mealtime effects of insulin may be largely salutary in brain, sustained hyperactivation of mammalian target of rapamycin (mTOR) by Akt, PKC-λ/ι, p70 S6K, or other factors^38^^,^^39^^,^^40^ can impair autophagy,^41^^,^^42^^,^^43^ which acts in conjunction with lysosomes to clear degenerating organelles and various macromolecules, and facilitate reuse of their constituents for cell repair.^44^ Additionally, sustained PKC-λ/ι hyperactivity in hyperinsulinemic states may be deleterious. In the brain,^34^ as in the liver,^45^^,^^46^ PKC-λ/ι phosphorylates and activates inhibitor of kappa-B kinase (IKK), which in turn phosphorylates the inhibitor of nuclear factor κB (IκB), thus releasing NF-κB for nuclear entry. This is followed by phosphorylation and activation of its p65RelA subunit that increases expression of both β-site amyloid precursor protein (βAPP) cleaving enzyme-1 (BACE1) and various proinflammatory factors, including cytokines, tumor necrosis factor α (TNF-α), interleukin 1β (IL-1β) and IL-6, that are thought to play a major pathogenetic role in AD.^47^ In turn, BACE1 increases Aβ production and Aβ-plaque formation, and is accompanied by increases in p-tau in hyperinsulinemic mice; moreover, all such brain alterations are blocked by variously acting chemical inhibitors of PKC-λ/ι and by heterozygous knockout (KO) of PKC-λ/ι.^32^^,^^33^^,^^34^

Several additional lines of evidence support the pathogenic potential of hyperinsulinemia. Firstly, Fishel et al.^31^ found that 1–2 h of hyperinsulinemia (in hyperinsulinemic-euglycemic clamp studies in normal humans) increases insulin levels in the cerebrospinal fluid (CSF) and is sufficient to provoke very sizable increases in CSF levels of Aβ peptides and pro-inflammatory cytokines (TNF-α, IL-1β, and IL-6). These findings demonstrate that hyperactivation of the insulin signaling system caused by peripherally derived insulin can drive AD-associated Aβ peptide formation and neuroinflammation in the CNS. Secondly, in transgenic (Tg) mouse models of AD, neuron-specific deletion of the insulin receptor^48^ or the IGF-1 receptor^49^ protects against AD development. Thirdly, increased activities of Akt, PKC-λ/ι, mTOR, and its co-target, p70 S6K, have repeatedly been seen in brains of humans with established AD, preclinical-AD, and amnestic mild cognitive impairment (MCI).^37^^,^^41^^,^^50^^,^^51^^,^^52^^,^^53^^,^^54^^,^^55^^,^^56^ Finally, autophagy, which is inhibited by mTOR, is dysfunctional or impaired in brains of humans with AD.^41^^,^^57^

Although MetS, T2DM, and hyperinsulinemia have received less attention as risk factors for FTD, several studies are suggestive.^29^^,^^30^ And the possibility that PGRN availability could directly perturb the insulin signaling pathway and influence neuropathology may be gleaned from reports showing that: (1) PGRN knockdown (KD) increases basal and insulin-stimulated IRS-1 activation (but not levels) in adipocytes^58^ and hepatocytes,^59^ without affecting insulin receptor (and therefore insulin-related IRS-2) activation; (2) IRS-1 variably activates PKC-λ/ι and/or Akt, depending upon the cell type (see the following section); and (3) PGRN has widespread anti-inflammatory and neuroprotective properties.^15^^,^^16^^,^^60^^,^^61^^,^^62^^,^^63^^,^^64^^,^^65^

Indeed, the similarity of the inhibitory effects of PGRN on Aβ-peptide and p-tau production in Tg AD mice^66^^,^^67^ to the effects of chemical inhibition or heterozygous KO of PKC-λ/ι in hyperinsulinemic MetS/T2DM mice^32^^,^^33^^,^^34^ led us to wonder if PGRN protects AD mice by decreasing insulin-regulated activity of PKC-λ/ι in the brain. However, relevant information is piecemeal in that: (1) whereas PGRN KD increases IRS-1 activation and Akt activity in adipocytes^58^ and IRS-1 controls aPKC as well as Akt in adipocytes,^68^ aPKC was not evaluated in this study; (2) whereas PGRN KD increases IRS-1 activation and Akt activity in hepatocytes,^59^ IRS-2, rather than IRS-1, controls hepatic aPKC^68^^,^^69^; and (3) whereas IRS-2 clearly controls Akt activation in brain,^70^^,^^71^^,^^72^^,^^73^^,^^74^ there is little or no direct information on IRS-1 regulation of Akt and PKC-λ/ι in brain.

Here, to determine if IRS-1 might link PGRN availability to the insulin signaling pathway in brain, and thus to NF-κB-dependent inflammation and mTOR-dependent autophagy dysfunction, we studied the effects of PGRN treatment and PGRN deficiency in both cultured human-derived neural and microglial cells, and in brains of mice with heterozygous and homozygous deletion of PGRN.

## Results

### Studies in human-derived LA1-5s neural and HMC3 microglial cells

#### Effects of PGRN on IRS-1 and IRS-2 signaling pathways in LA1-5s neural cells

**Figure 1:**
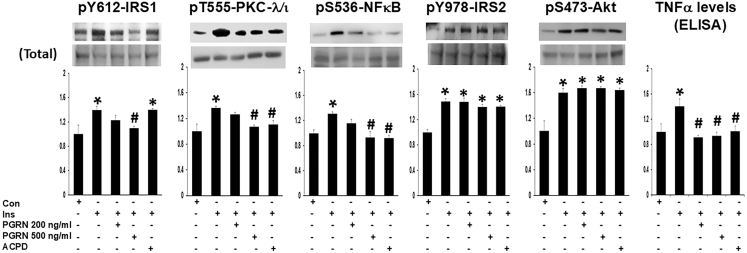
Progranulin inhibits insulin-induced increases in activation of IRS-1, PKC-λ/ι, and NF-κB, and levels of TNF-α, without altering IRS-2 and Akt activation in LA1-5s neural cells Cells were incubated for 24 h as untreated controls (Con) or treated with 200 nM insulin (Ins) ± 200 ng/mL or 500 ng/mL PGRN or 1 μM PKC-λ/ι inhibitor, ACPD. Values in bar graphs are mean ± SEM of 4 comparisons of findings in treated samples relative to findings in untreated control (Con) samples, with the latter set as unity. Asterisks (∗) indicate a significant difference of *p* < 0.05 (ANOVA) between treated versus untreated control samples. Pound signs (#) indicate a significant difference (*p* < 0.05) between insulin plus PGRN or insulin plus ACPD treatment, versus treatment with insulin alone. S, serine; T, threonine; Y, tyrosine.

It was previously shown that insulin acts via PKC-λ/ι to activate both IKKα/β and NF-κB and subsequently increase mRNA and protein and levels of BACE1 and proinflammatory cytokines in human-derived LA1-5s neural cells.^34^ Presently, we showed that insulin activates both IRS-1 and IRS-2 in these cells, as surmised from increases in phospho-tyr-612-IRS-1 (pY-612-IRS-1) and phospho-tyr-978-IRS-2 (pY-978-IRS-2) coupled with activation of downstream protein kinases, PKC-λ/ι and Akt (Figure 1). Next, we questioned whether PGRN neuroprotection may involve decreases in the activity of PKC-λ/ι and its activation of the IKK/NF-κB proinflammatory pathway, and we indeed found that PGRN (in concentrations comparable to those used to diminish BACE1 expression in N2a neural cells, or activate phagocytosis in mouse microglial cells,^67^ or inhibit IRS-1 activation in adipocytes^58^ and hepatocytes^59^) blocked insulin-induced increases in activation of IRS-1, activities of PKC-ι/λ and NF-κB, and associated increases in levels of TNF-α, but had no appreciable effect on the activation of IRS-2 and Akt in LA1-5s neural cells (Figure 1). And, like PGRN, aPKC inhibitor 2-acetyl-cyclopentane-1,3-diketone (ACPD) blocked insulin effects on activities of PKC-λ/ι and NF-κB, and levels of TNF-α, but, unlike PGRN, did not inhibit IRS-1 activation, i.e., insulin sensitive factors upstream of PKC-λ/ι (Figure 1). Further note that in the present conditions, total levels of IRS-1, PKC-λ/ι , and NF-κB (lower bands, Figure 1) were not altered by insulin, PGRN and ACPD treatments, and increases in the phosphorylation of the key Ser and Thr activation sites shown in Figure 1 (also reviewed in STAR Methods) reflect increases in the activation and/or specific activities of the these signaling factors. It may therefore be surmised that, in LA1-5s neural cells, in the main: (1) IRS-1 mediates insulin activation of PKC-λ/ι; (2) PKC-λ/ι controls NF-κB activation and subsequent increases in TNF-α; (3) insulin activates Akt largely independently of IRS-1, most likely via IRS-2, as suggested by findings in brain IRS-2 KO studies^70^^,^^71^^,^^72^^,^^73^^,^^74^; and (4) PGRN inhibits IRS-1 activation and thereby inhibits PKC-λ/ι and NF-κB activation.

#### Effects of PGRN on IRS-1 and IRS-2 signaling pathways in HMC3 microglial cells

**Figure 2:**
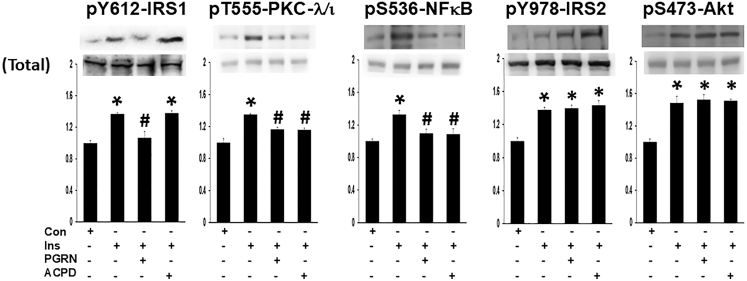
Progranulin inhibits insulin-induced increases in activation of IRS-1, PKC-λ/ι, and NF-κB, without altering IRS-2 and Akt activation in HMC3 microglial cells Cells were incubated for 24 h as untreated controls (Con) or treated with 200 nM insulin (Ins) ± 500 ng/mL PGRN or 1 μM PKC-λ/ι inhibitor, ACPD. Values in bar graphs are mean ± SEM of 4 comparisons of findings in treated samples relative to findings in untreated control (Con) samples, with the latter set as unity. Asterisks indicate a significant difference of *p* < 0.05 (ANOVA) between findings in treated samples versus findings in untreated samples. Pound signs (#) indicate a significant difference (*p* < 0.05) between insulin plus PGRN or insulin plus ACPD treatment versus treatment with insulin alone.

Similarly, in human-derived HMC3 microglial cells: (1) PGRN blocked insulin-induced activation of IRS-1, PKC-λ/ι, and NF-κB but had no appreciable effect on IRS-2 or Akt activation (as in Figure 1, all enzyme activities in Figure 2 are indicated by the phosphorylation of key sites); (2) aPKC inhibitor ACPD blocked insulin activation of PKC-λ/ι and NF-κB, but had no effect on IRS-1 activation; and (3) levels of IRS-1, PKC-λ/ι, and NF-κB were unchanged by 24 h treatment with insulin, PGRN and ACPD (see lower bands, Figure 2) (accordingly, the above-cited alterations in key enzyme-activating phosphorylation sites reflect changes in specific enzyme activity). Thus, during insulin action in both LA1-5s neural and HMC3 microglial cells, it appears that IRS-1 functions upstream of PKC-λ/ι, PKC-λ/ι functions upstream of NF-κB, and Akt largely functions independently of IRS-1, most likely downstream of IRS-2.

#### Effects of deletion of endogenous PGRN on IRS-1 and IRS-2 signaling pathways in HMC3 cells

**Figure 3:**
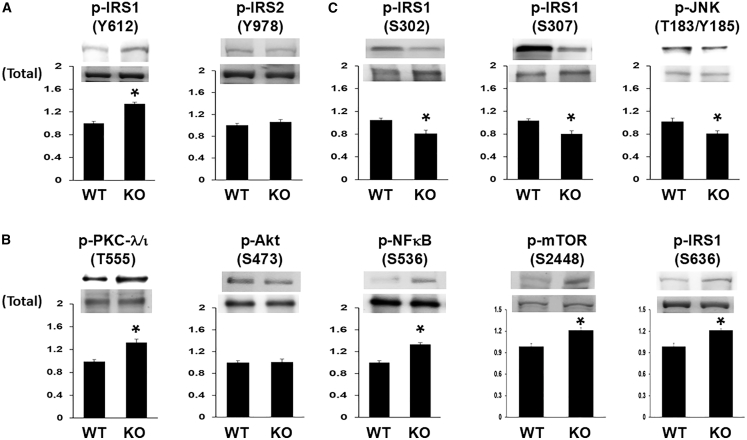
Progranulin deletion in HMC3 microglial cells increases activation of IRS-1, PKC-λ/ι, NF-κB, and mTOR PGRN KO HMC3 cells have: (A) increased activation of IRS-1, but not of IRS-2; (B) increased activities of PKC-λ/ι, NF-κB and mTOR, but not of Akt; and (C) diminished activity of JNK and phosphorylation of Ser-302 and Ser-307 of IRS-1. Values in bar graphs are mean ± SEM of 4 comparisons of findings in PGRN KO cells relative to findings in wild-type (WT) cells, with the latter set as unity. Asterisks indicate a significant difference of *p* < 0.05 (ANOVA) between findings in PGRN KO cells versus findings in WT cells. Figure S1 shows the loss of PGRN and the increases in mRNA levels of TNF-α, IL-1β, and IL-6 in PGRN KO cells.

As discussed, short hairpin RNA (shRNA)-induced KD of PGRN increases basal/resting IRS-1 activation in mouse adipocytes^58^ and hepatocytes,^59^ and IRS-1 in turn activates Akt in mouse adipocytes and hepatocytes. Here, to diminish PGRN levels in HMC3 microglial cells, PGRN was knocked out (see PGRN loss in Figure S1) by CRISPR-Cas9 methods and PGRN KO cells were compared to wild-type (WT) cells. As with PGRN KD in adipocytes^58^ and hepatocytes,^59^ PGRN KO in HMC3 cells led to increases in basal/resting activation of IRS-1 (as per increases in key enzyme-activating phosphorylation sites, without affecting levels of IRS-1), and had no discernable effect on IRS-2 activation or levels (Figure 3A). On the contrary, whereas PGRN-deficiency-induced IRS-1 activation in HMC3 microglial cells led to increases in basal/resting activity (as per changes in key phosphorylations, but not levels) of PKC-λ/ι and NF-κB, the activity and levels of Akt were not appreciably altered (Figure 3B). Moreover, despite unaltered Akt activity, mTOR phosphorylation/activity was elevated in HMC3 PGRN KO cells and accompanied by increases in phosphorylation of Ser-636/639-IRS-1, a target of mTOR-dependent p70 S6 kinase (S6K)^75^ (Figure 3B). The latter finding presumably reflects that mTOR and S6K are co-activated by factors other than Akt,^38^^,^^40^ including aPKC^39^; indeed, mTOR was found to be activated along with aPKC in livers of MetS/T2DM humans despite diminished Akt activity.^76^ Finally note, as in LA1-5s neural cells,^34^ increases in expression (as per mRNA levels) of proinflammatory cytokines (TNF-α, IL-1β, and IL-6) accompanied the increases in PKC-λ/ι and NF-κB activity that ensued from PGRN KO in HMC3 microglial cells (Figure S1).

#### Effects of PGRN deletion on JNK activity and phosphorylation of Ser residues of IRS-1 in HMC3 cells

It seems clear that phosphorylation of Ser-302 and Ser-307 residues of IRS-1 serve as important negative regulators of IRS-1^75^^,^^77^^,^^78^ and the phosphorylation of both Ser residues is increased by JNK and co-required for its negative effects on insulin-stimulated IRS-1 activation^77^; moreover, JNK and Ser-307-IRS-1 phosphorylation have been found to be involved in the negative regulation of IRS-1 activation by PGRN in mouse adipocytes.^79^ Accordingly, we questioned whether JNK-regulated phosphorylation of Ser residues in IRS-1 might underlie PGRN-dependent alterations in basal IRS-1 activation in HMC3 microglial cells. Indeed, along with PGRN-KO-induced increases of basal IRS-1 activation (and unchanged IRS-1 levels) in HMC3 cells, there were decreases in basal JNK activity (but not levels), accompanied by decreases in the phosphorylation of Ser-302-IRS-1 and Ser-307-IRS-1 (Figure 3C); in contrast, the phosphorylation of Ser-616-IRS-1 was unaltered (not shown) and the phosphorylation of Ser-636/639-IRS-1 was increased by PGRN KO (Figure 3B), presumably owing to IRS-1 activation of PKC-λ/ι and mTOR, as discussed earlier.

#### Effects of PGRN on JNK activity and phosphorylation of Ser residues of IRS-1 in HMC3 cells

**Figure 4:**
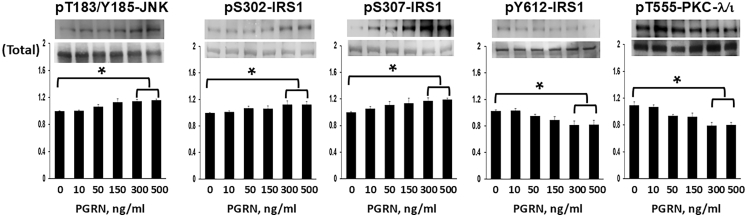
Progranulin increases JNK activation and IRS-1 phosphorylation at Ser-302 and Ser-307 PGRN increases the activation of JNK and the phosphorylation of Ser-302-IRS-1 and Ser-307-IRS-1, and concomitantly decreases activation of IRS-1 and PKC-λ/ι in HMC3 microglial cells. Cells were incubated for 24 h with indicated concentrations of PGRN. Values in bar graphs are mean ± SEM of 4 comparisons of findings in PGRN-treated cells relative to findings in untreated control cells, with the latter set as unity. Asterisks indicate a significant difference of *p* < 0.05 (ANOVA) between findings in cells treated with maximally effective PGRN concentrations versus findings in untreated control cells.

In concert with the idea that JNK-induced increases in phosphorylation of Ser-302 and Ser-307 residues might contribute to the negative regulation of basal IRS-1 activation by PGRN in HMC3 cells, we found that 24 h PGRN treatment increased both JNK activity (not levels) and the phosphorylation of inhibitory Ser-302 and Ser-307 residues of IRS-1, and concomitantly decreased IRS-1 activation and PKC-λ/ι activity (but not levels) in HMC3 cells (Figure 4). Note that total IRS-1 levels are shown in Figure 4 and, on average, there were no significant changes; whether longer periods of PGRN treatment would lead to decreased IRS-1 levels, as might be expected, was not presently studied.

#### Effects of JNK inhibitor on Ser-307/302 phosphorylation and IRS-1 activation in HMC3 cells

**Figure 5:**
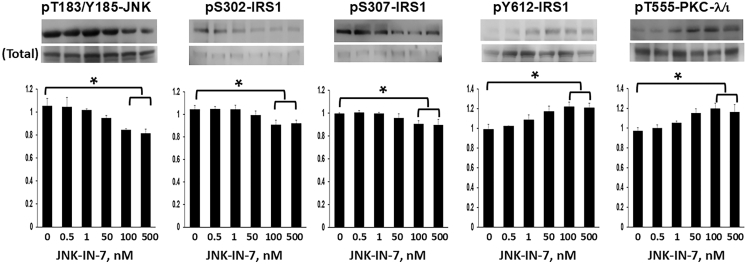
JNK inhibitor, JNK-IN-7, diminishes JNK activity and its phosphorylation of Ser-302 and Ser-307 of IRS-1, and concomitantly increases IRS-1 activation and PKC-λ/ι activity in HMC3 microglial cells Cells were incubated for 24 h with indicated concentrations of JNK-IN-7. Values in bar graphs are mean ± SEM of 4 comparisons of findings in JNK-IN-7-treated cells relative to findings in untreated control cells, with the latter set as unity. Asterisks indicate a significant difference of *p* < 0.05 (ANOVA) between findings in cells treated with maximally effective JNK-IN-7 concentrations versus findings in untreated control cells.

In keeping with the idea that JNK is involved in restraining basal IRS-1 activation during the action of endogenous PGRN, we found in WT HMC3 cells that 24 h treatment with JNK-IN-7, an inhibitor of JNK1, JNK2 and brain-specific JNK3,^80^^,^^81^ diminished JNK activity (but not levels) and the phosphorylation of Ser-302-IRS-1 and Ser-307-IRS-1, and concomitantly increased the basal/resting activation (but not levels) of IRS-1 and activity of PKC-λ/ι (Figure 5). In contrast, JNK-IN-7 had no appreciable effect on IRS-1 activation and PKC-λ/ι activity in PGRN KO cells, (Figure S2) suggesting that the increases in IRS-1 activation and PKC-λ/ι activity seen in JNK-IN-7-treated WT cells reflected decreases in activity of endogenous PGRN.

### Studies of PGRN KO and WT mice

#### Effects of PGRN deficiency on insulin signaling factors in mouse tissues outside of brain

As discussed, it was previously shown that (1) acute KD-induced or chronic KO-induced PGRN deficiency in mouse adipocytes and adipose tissue^58^ and mouse hepatocytes^59^ leads to increases in IRS-1 activation and Akt activity (as per respective pY-612 and pSer-473 contents), without changes in their levels, and without changes in levels or activation of the insulin receptor; (2) PGRN treatment increases JNK activity and phosphorylation of Ser-307-IRS-1 in mouse adipose tissue^79^; (3) both Akt and aPKC are controlled by IRS-1 during insulin action in mouse adipose tissue and muscle^68^; and (4) Akt functions mainly downstream of IRS-1 and aPKC functions downstream of IRS-2 (not IRS-1) in mouse^68^^,^^69^ and human^82^ liver. In concert with these prior observations, we found that heterozygous and homozygous deletion of PGRN in mice led to decreases in JNK activity and phosphorylation of Ser-302-IRS-1 and Ser-307-IRS-1, not only in adipose tissue but also in all tissues examined (including three major sites of insulin action, viz., fat, liver, and muscle) and all were coupled with reciprocal increases in IRS-1 activation. And, as might be expected,^68^^,^^69^ IRS-1 activation led to Akt activation in fat, liver, and muscle, and to aPKC activation in fat and muscle, but not in liver, where IRS-2 controls aPKC activation^68^^,^^69^ (Figure S3).

#### Effects of PGRN deficiency on insulin signaling factors in mouse brain

**Figure 6:**
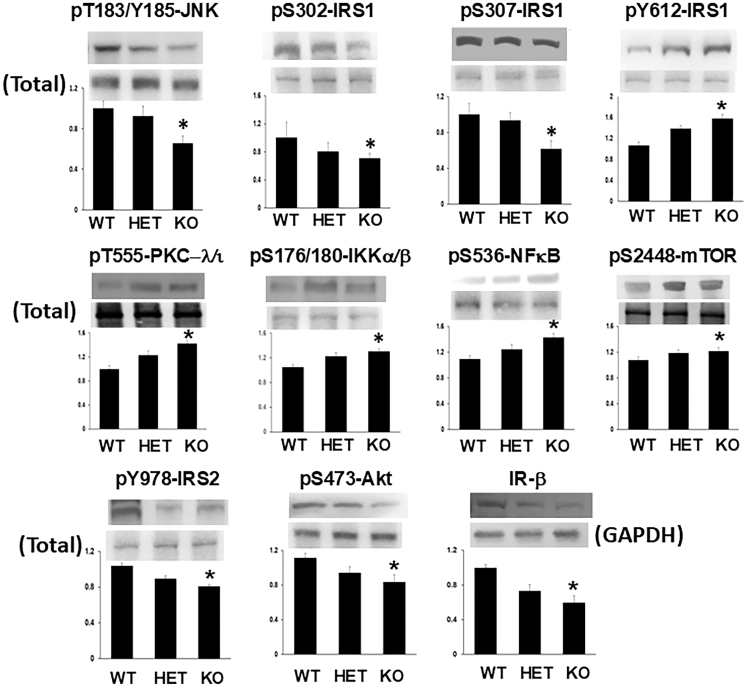
Progranulin deficiency in mouse brain diminishes activity of JNK and phosphorylation of Ser-302 and Ser-307 of IRS-1, and concomitantly increases activation of IRS-1 and activities of PKC-λ/ι, IKKα/β, NF-κB, and mTOR In brain cortical tissues of 2- to 4-month-old male and female mice, heterozygous (HET) and homozygous knockout (KO) of progranulin (PGRN) (1) diminishes activity of JNK and phosphorylation of Ser-302-IRS-1 and Ser-307-IRS-1, and (2) concomitantly increases activation of IRS-1 and activities of PKC-λ/ι, IKKα/β, NF-κB, and mTOR, while (3) diminishing IRS-2 activation, Akt activity, and IR-β levels. Values in bar graphs are mean ± SEM of 4 comparisons of findings in HET or KO mice relative to findings in wild-type (WT) mice, with the latter WT mean values set as unity. As findings were comparable in male and female mice, results were combined. Asterisks indicate a significant difference of *p* < 0.05 (ANOVA) between findings in KO and WT mice.

As discussed previously, whereas it is clear that brain Akt activity is in large part controlled by IRS-2,^70^^,^^71^^,^^72^^,^^73^^,^^74^^,^^83^ there is little or no direct information on the role of IRS-1 in the control of either Akt, or PKC-λ/ι in the brain. Presently, as in peripheral tissues, we found in mouse brain that heterozygous and homozygous KO of PGRN led to graded decreases in PGRN levels (Figure S4) that were accompanied by decreases in activity but not levels of JNK, in turn accompanied by decreases in phosphorylation of Ser-302-IRS-1 and Ser-307-IRS-1, and by associated increases in IRS-1 activation, but not levels (Figure 6). But unlike peripheral tissues, increases in IRS-1 activation in brains of PGRN KO mice were accompanied by increases in activity of both PKC-λ/ι and NF-κB, but, surprisingly, not Akt, whose activity, like that of IRS-2, was diminished (Figure 6). Of further note, in brains of PGRN KO mice (1) decreases in IRS-2 activation and Akt activity were accompanied by decreases in the levels of the insulin receptor β-subunit (IR-β); (2) despite decreases in Akt activity, the activity of mTOR was increased, presumably owing to increases in PKC-λ/ι activity^38^^,^^39^^,^^40^; and (3) total levels of JNK, IRS-1, IRS-2, Akt, PKC-λ/ι, IKKα/β, NF-κB, and mTOR were unchanged by PGRN KO (Figure 6).

## Discussion

### Widespread action of PGRN on JNK and inhibition of basal IRS-1 activation

The present findings show that mice with reduced PGRN levels have increased basal/resting activation of IRS-1 in all examined tissues, including major tissues targeted by insulin, viz., white adipose tissue, liver, hindlimb skeletal muscle and brain cortex, and these increases in IRS-1 activation showed a gene-dosage relationship, with stronger effects in homozygous vis-a-vis heterozygous KO mice. Similarly, in isolated, human-derived, PGRN-rich HMC3 microglial cells, basal IRS-1 activation was increased by CRISPR-Cas9-mediated PGRN deletion and decreased by PGRN treatment. Of further note, all PGRN-associated changes in IRS-1 activation in mouse tissues and cultured microglial cells were accompanied by, and most likely resulted from, reciprocal alterations in JNK activity and phosphorylation of Ser-302-IRS-1 and Ser-307-IRS-1, which are co-phosphorylated by JNK and co-required for JNK-mediated inhibition of basal and insulin-stimulated IRS-1 activation.^75^^,^^77^^,^^78^^,^^84^ Indeed, in HMC3 microglial cells: (1) PGRN treatment activated JNK and increased Ser-302-IRS-1 and Ser-307-IRS-1 phosphorylation while decreasing basal IRS-1 activation; and (2) oppositely, treatment with a JNK inhibitor diminished basal/resting JNK activity and phosphorylation of Ser-302-IRS-1 and Ser-307-IRS-1, while increasing basal IRS-1 activation. Moreover, in both mouse brain cortex and human-derived neural and microglial cells, all PGRN-related alterations in IRS-1 activation led to parallel changes in PKC-λ/ι, but not IRS-2 activation or Akt activity.

Our findings therefore suggest that, in the normal basal/resting state, in multiple (all tested) tissues, PGRN acts constitutively via JNK to phosphorylate Ser-302-IRS-1 and Ser-307-IRS-1 and thereby reduce basal/resting IRS-1 activation; and, oppositely, with PGRN deficiency, decreases in JNK activity and phosphorylation of Ser-302-IRS-1 and Ser-307-IRS-1 lead to increases in basal/resting IRS-1 activation and associated changes in activity of Akt and/or aPKC in a tissue-specific manner.

### Metabolic effects of PGRN regulation of IRS-1 activation outside of the brain

It appears that PGRN deficiency and ensuing increases in IRS-1 activation in adipose tissue, liver, and muscle would most likely enhance insulin-regulated glucose metabolism and diminish MetS/T2DM development, as (1) in adipose tissue and skeletal muscle, Akt and aPKC are co-dependent on IRS-1 for activation^68^ and co-required for insulin stimulation of glucose transport^85^; and (2) in liver, whereas Akt is mainly IRS-1 dependent^68^^,^^69^ and is a major mediator of inhibitory effects of insulin on hepatic glucose production and release, hepatic aPKC activation by insulin is IRS-2 rather than IRS-1-dependent^68^^,^^69^ and moreover impairs IRS-1-dependent activation of hepatic Akt and its salutary inhibitory effects on gluconeogenic enzyme expression.^85^^,^^86^^,^^87^^,^^88^

Accordingly, the alterations in Akt and aPKC that follow IRS-1 activation in adipose tissue, liver, and muscle could help explain how (1) PGRN deficiency protects mice from developing glucose intolerance and insulin resistance in response to high-fat feeding^58^; and (2) PGRN excess/treatment enhances MetS/T2DM development in mice.^59^ In the latter regard, it is interesting to note that PGRN blood levels are elevated in ob/ob mice^58^ and humans with MetS^89^ and T2DM.^90^

### Effects of PGRN regulation of IRS-1 activation in brain

**Figure 7:**
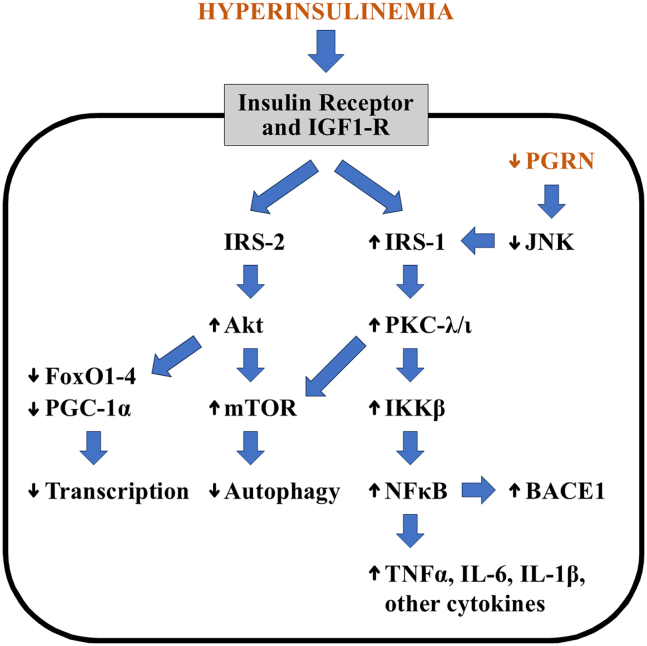
Activation of the insulin signaling system in brain by hyperinsulinemia and progranulin (PGRN) deficiency and sequential increases in activation of insulin receptor substrate-1 (IRS-1) and activities of PKC-λ/ι, inhibitor of kappa-B kinase (IKK) and nuclear factor-kB (NF-κB), followed by increases in expression of NFκB-dependent BACE1 and proinflammatory factors, TNF-α, IL-6, and IL-1β Hyperinsulinemia also activates IRS-2 and Akt, which along with PKC-λ/ι, increases mTOR activity, which leads to impairments in autophagy and lysosomal activity.

But opposite to the unsalutary effects of PGRN excess/treatment and salutary effects of PGRN deficiency on glucose metabolism that may derive from alterations in IRS-1 activation in adipose tissue, liver, and muscle, the IRS-1-related effects of PGRN excess and PGRN deficiency in brain are likely to be largely salutary and unsalutary, respectively. Thus, PGRN excess/treatment, by inhibiting brain IRS-1, PKC-λ/ι, IKKα/β, NF-κB, and mTOR, could contribute to neuroprotective effects of PGRN noted generally^15^^,^^16^^,^^60^^,^^61^^,^^62^^,^^63^^,^^64^^,^^65^ and more specifically in mouse models of FTD,^91^^,^^92^ neuronal ceroid lipofuscinosis^93^ and AD.^66^^,^^67^^,^^94^ Oppositely, as portrayed in Figure 7, PGRN deficiency, by activating brain IRS-1 and PKC-λ/ι, may enhance neuropathology development by: (1) increasing NF-κB activity and expression of both proinflammatory cytokines^34^ and BACE1,^34^^,^^95^^,^^96^^,^^97^ which increases Aβ-peptide production and Aβ-plaque formation; (2) increasing tau phosphorylation^32^^,^^33^^,^^34^ perhaps via increases in mTOR/p70-S6K^50^^,^^51^^,^^52^^,^^53^; and (3) increasing mTOR activity and thereby impairing autophagy and lysosomal biogenesis and function.^42^^,^^43^^,^^44^

It was of course surprising to find that opposite to IRS-1-dependent increases in Akt activity in adipose, muscle, and liver tissues of PGRN-deficient mice, Akt activity was reduced in the mouse brain cortex despite increases in IRS-1 activation; furthermore, Akt activity in both mouse brain cortex and isolated neural and microglial cells correlated with changes in IRS-2, rather than IRS-1, activation. Accordingly, it might be construed that insulin activation of brain Akt is uniformly independent of IRS-1 and mainly controlled by IRS-2, i.e., coincident with findings in multiple IRS-2 KO studies.^70^^,^^71^^,^^72^^,^^73^^,^^74^^,^^83^ On the contrary, (1) there are three or more Akt isoforms and less abundant isoforms may have escaped detection in our analyses, and (2), the brain is very heterogeneous in cell types and functional areas, and IRS-1 and IRS-2 may have different functions therein. Additionally, alterations in activation or function of IRS-1 or other PGRN-related factors may have led to compensatory alterations in the control of Akt activation by IRS-1, IRS-2, or other less studied forms of IRS.

Regardless of the cause, the reductions in IRS-2 activation and Akt activity in brains of PGRN-deficient mice may in part reflect the decreases in IR-β levels that were concomitantly seen in these brains. In keeping with this idea, in livers and brains of high-fat-fed hyperinsulinemic mice, we found that decreases in IR-β levels appeared to follow PKC-λ/ι-dependent increases in BACE1,^98^ which proteolytically degrades IR-β.^99^ Although further studies are needed to see if this mechanism is operative in human dementias, note that decreased insulin receptor levels have been observed in brains of T2DM humans with cognitive impairment,^100^ presumably an AD precursor.

Further note that decreases in IRS-2 activation and Akt activity in brains of PGRN-deficient mice may have unsalutary effects by: (1) decreasing glucose transport in glial cells, and impairing glial-dependent delivery of glycolytic energy-producing metabolites to neurons^101^; (2) diminishing activities of eukaryotic initiation factor 4-E,^102^ which is needed to initiate protein synthesis; (3) decreasing phosphorylation and activity of various FoxOs that have anti-apoptotic effects^103^; and (4) decreasing phosphorylation and increasing activity of glycogen synthase kinase-3β, which can increase tau phosphorylation^104^; and (5) impairing long-term potentiation (LTP) and dependent cortical functions.^73^^,^^74^

### Comparison of alterations in brain insulin signaling in various dementias and their risk factors

It may be noted that the reduction of Akt activity resulting from impaired IRS-2 activation in brains of PGRN-deficient mice differs from increases in Akt activity seen in brains of (1) humans with AD and its forerunners, pre-clinical AD, and MCI^37^^,^^41^^,^^52^^,^^55^ and (2) hyperinsulinemic MetS/T2DM mice and monkeys.^32^^,^^33^^,^^34^ Moreover, the combined activation of Akt and PKC-λ/ι in AD brain^37^ may provide ready explanation for the increases in mTOR activity repeatedly seen in AD brain^37^^,^^41^^,^^50^^,^^52^^,^^53^^,^^55^ and thought to lead to impairments in autophagy and lysosomal function.^41^^,^^57^ Nevertheless, despite diminished Akt activity in PGRN-deficient brain, elevations in mTOR activity were readily seen and most likely reflective of increases in PKC-λ/ι activity, as discussed previously. In any case, regardless of how mTOR activity is increased, the combination of PKC-λ/ι-dependent activation of the NF-κB-dependent inflammatory pathway and PKC-λ/ι ± Akt dependent activation of mTOR and its impairment of autophagy and lysosomal function may serve as complementary pathogenetic mechanisms that are common to FTD and AD.

In comparing the present findings in PGRN deficient mice, as a model of FTD, to those seen in other mouse models of dementia, it is interesting to note that IRS-1 levels are increased in hippocampal areas of Tg APP/PS1 AD mice, and, moreover, accompanied by decreased levels of IRS-2, Akt, and *p*-Ser-473-Akt,^105^^,^^106^ i.e., net changes in insulin signaling remarkably similar to those presently seen in brains of PGRN-deficient mice. Of further note, treatment with curcumin, which is thought to be neuroprotective in AD, reversed IRS-1 increases and IRS-2 decreases and simultaneously improved glucose uptake (as per positron emission tomography [PET] scans) in various brain areas of Tg APP/PS1 AD mice.^106^

### Limitations of the study

We presently did not evaluate changes in specific areas of the brain or behavioral consequences at various time points of PGRN deficiency. Future studies of these parameters, as well as additional validation of our *in vivo* findings, are needed. Further studies are also needed to determine the mechanism for JNK activation that results from PGRN deficiency; and, in this regard, note that JNK (aka stress-activated protein kinase) isoforms, JNK1, JNK2, and JNK3, are phosphorylated and activated by mitogen-activated protein kinases (MAPKKs) and MAPKKKs that are variously controlled by (1) growth factors, including, insulin^107^^,^^108^ and ephrins acting via ephrin tyrosine kinase receptor-A2 (EphA2),^109^ which is postulated to serve as a PGRN receptor^110^ and (2) stress factors, including TNF-α,^75^^,^^111^^,^^112^^,^^113^ which was reported to act via a receptor shared with PGRN.^114^

Further studies are also needed to evaluate the likelihood that the restraint of IRS-1 by PGRN and JNK serves protectively. For example, by activating JNK and limiting the ability of IRS-1 to diminish hepatic glucose production while at the same time permitting hepatic IRS-2 and PKC-λ/ι to raise blood glucose levels, PGRN may diminish the likelihood of developing hypoglycemia during periods of inanition. Of particular importance in nonregenerative brain, PGRN may have beneficial protective effects by activating JNK and restraining untoward activation of the IRS-1/PKC-λ/ι/IKK/NF-κB/mTOR pathway, thus limiting NFκB-dependent inflammation and mTOR-dependent impairment of autophagy and lysosome biogenesis. In this regard, it is interesting to note that overall JNK activity is decreased in brains of humans with FTD, but not other dementias,^115^ wherein JNK activation seemingly follows (rather than precedes) neuroinflammation and neurodegeneration.

While our results clearly point to a JNK-dependent, presumably protective mechanism in which PGRN restrains IRS-1, PKC-λ/ι, IKK, NF-κB, and mTOR signaling in the brain, we presently can only speculate that aberrations in JNK and the insulin signaling pathway contribute importantly to neuropathology development in PGRN-deficient states, including, FTD,^2^^,^^3^^,^^4^ lipofuscinosis,^116^^,^^117^ and certain forms of AD with PGRN mutations and non-coding polymorphisms.^5^^,^^6^^,^^7^^,^^8^^,^^9^^,^^118^^,^^119^^,^^120^ And, with respect to FTD, further studies are needed to determine (1) whether the presently observed stimulatory effects of PGRN deficiency on the brain IRS-1/PKC-λ/ι/NF-κB/mTOR insulin signaling pathway and the widespread impairments in autophagy and lysosomal function seen in PGRN-deficient FTD^121^^,^^122^^,^^123^^,^^124^ are causally interrelated, or independent, but complementary, pathogenetic mechanisms and (2) whether there are similar alterations in the insulin signaling pathway in non-PGRN-related forms of FTD, e.g., FTD associated with C9orf72 hexanucleotide repeats, microtubule-associated p-tau (*MAPT*) mutations, or other genetic abnormalities, and sporadic FTD,^125^^,^^126^ and forms of AD with associated *GRN* mutations and polymorphisms.^5^^,^^6^^,^^7^^,^^8^^,^^9^ Finally, note that there is an urgent need to see if deficiencies of PGRN or other genetic factors thought to cause human FTD lead to increases in IRS-1 activation and activities of PKC- λ/ι, IKKα/β, NF-κB, and/or decreases in IRS-2 and Akt in brains of humans with FTD that were presently observed in PGRN deficient mouse brain.

## Resource availability

### Lead contact

Further information and requests for resources and reagents should be directed to and will be fulfilled by the lead contact, Robert V. Farese (rfarese@usf.edu).

### Materials availability

Materials are made available upon request.

### Data and code availability

• All data generated in this study are included in the article.
• This paper does not report original code.
• Any additional information required to reanalyze the data reported in this paper is available from the lead contact upon request.

## Acknowledgments

We thank the Genome Engineering and Stem Cell Center at Washington University for assistance with generating PGRN KO cells.

This work was supported by grants from the 10.13039/100000002National Institutes of Health (NIH) [AG047339] and by the Washington University Institute of Clinical and Translational Sciences grant [UL1TR002345] from the NIH 10.13039/100006108National Center for Advancing Translational Sciences (NCATS). Funding was also provided by donations from the Kaul Foundation, Tampa, Florida to the USF Foundation. The content is solely the responsibility of the authors and does not necessarily represent the official views of the NIH and the Veterans Administration.

## Author contributions

R.V.F. and A.D.N. conceived and provided overall direction to the studies; M.P.S. conducted western blot and ELISA assays and analyzed these data; B.C.H. and M.A.D. assisted in these assays; D.R.C. performed experiments in LA1-5s cells; G.A., J.J.H., and D.M.S. performed studies in HMC3 cells and PGRN KO mice and conducted mRNA analyses.

## Declaration of interests

The University of South Florida has patents on the use of aPKC inhibitors for neurodegenerative disorders.

## STAR★Methods

### Key resources table

**Table:** 

REAGENT or RESOURCE	SOURCE	IDENTIFIER
**Antibodies**		

Rabbit monoclonal anti-IR-β	Cell Signaling	Cat #3025; RRID: AB_2280448
Rabbit polyclonal anti-*p*-tyr-612-IRS-1	Cell Signaling	Cat #2386; RRID: AB_330326
Rabbit polyclonal anti-*p*-ser-302-IRS-1	Cell Signaling	Cat #2384; RRID: AB_330360
Rabbit polyclonal anti-*p*-ser-307-IRS-1	Cell Signaling	Cat#2381; RRID: AB_330342
Rabbit polyclonal anti-*p*-ser-616-IRS-1	Invitrogen/Thermo Fisher	Cat#44-550-G; RRID: AB_1501245
Rabbit polyclonal anti-*p*-ser-636/639-IRS-1	Cell Signaling	Cat#2388; RRID: AB_330339
Rabbit polyclonal anti-IRS-1	Cell Signaling	Cat#2382; RRID: AB_330333
Rabbit polyclonal anti-*p*-tyr-978-IRS-2	MyBioSource	Cat#9211379
Rabbit polyclonal anti-IRS-2	Cell Signaling	Cat#4502; RRID: AB_2125774
Rabbit polyclonal anti-*p*-ser-473-Akt	Cell Signaling	Cat#9271; RRID: AB_329825
Rabbit polyclonal anti-Akt	Cell Signaling	Cat#9272; RRID: AB_329827
Rabbit polyclonal anti-*p*-thr-555-PKC-λ/ι	Invitrogen/Thermo Fisher	Cat#44-968-G; RRID: AB_1501968
Mouse monoclonal anti-PKC-λ/ι	Santa Cruz	Cat#sc-376344; RRID: AB_10988253
Rabbit monoclonal anti-*p*-ser-176/180-IKKα/β	Cell Signaling	Cat#2697; RRID: AB_2079382
Rabbit monoclonal anti-IKK	Cell Signaling	Cat#8943; RRID: AB_11024092
Rabbit monoclonal anti-*p*-ser-529/536-NFκB/p65RelA	Cell Signaling	Cat#3033; RRID: AB_331284
Rabbit monoclonal anti-NFκB/p65RelA	Cell Signaling	Cat#8242; RRID: AB_10859369
Rabbit polyclonal anti-*p*-ser-2448-mTOR	Cell Signaling	Cat#2971; RRID: AB_330970
Rabbit polyclonal anti-mTOR	Cell Signaling	Cat#2972; RRID: AB_330978
Rabbit polyclonal anti-BACE1	Invitrogen/Thermo Fisher	Cat#PA1-757; RRID: AB_325863
Rabbit polyclonal anti-*p*-thr-183/p-tyr-185-JNK	Cell Signaling	Cat#9251; RRID: AB_331659
Rabbit polyclonal anti-JNK	Cell Signaling	Cat#9252; RRID: AB_2250373
Rabbit polyclonal anti-human progranulin	Nguyen et al.^127^	N/A
Sheep polyclonal anti-mouse progranulin	R&D Systems	Cat#AF2557; RRID: AB_2114504
Rabbit monoclonal anti-β-Actin	Cell Signaling	Cat#8457; RRID: AB_10950489
Rabbit monoclonal anti-Vinculin	Cell Signaling	Cat#13901; RRID: AB_2728768

**Chemicals, peptides, and recombinant proteins**		

Recombinant insulin (human)	Sigma-Aldrich	Cat#I9278
Recombinant progranulin (human)	R&D Systems	Cat#2420-PG-50
2-acetyl-cyclopentane-1,3-diketone (ACPD)	Sigma-Aldrich	Cat#R426911
JNK-IN-7	Cayman Chemicals	Cat#28831

**Experimental models: Cell lines**		

Human: LA1-5s neural cells	European Collection of Authenticated Cells	Cat#06041204; RRID: CVCL_2549
Human: HMC3 microglial cells	American Type Culture Collection	Cat#CRL-3304; RRID: CVCL_II76

**Experimental models: Organisms/strains**		

Mouse: *Grn*^−/−^ mice	Martens et al.^15^	RRID: MMRRC_036771-JAX

**Oligonucleotides**		

Primers for qPCR, see Table S1	This paper	N/A
gRNA sequences for CRISPR/Cas9: CCCTGAGACGGTAAAGATGCNGG and CCTGCATCTTTACCGTCTCANGG	This paper	N/A

### Experimental models and study participant details

#### Cell models

LA1-5s neural cells (obtained from European Collection of Authenticated Cells, ECACC, 06041204) were originally derived from a human neuroblastoma, and cultured and used in experiments as described.^32^^,^^33^^,^^34^ HMC3 human microglial cells (obtained from the American Type Culture Collection, ATCC, CRL-3304) were cultured in Eagle’s Minimum Essential Medium (EMEM) (Corning, 10-009-CV) supplemented with 10% fetal bovine serum (FBS) (Gibco, 26140–095), 10U/mL penicillin, and 10 μg/mL streptomycin (Gibco, 15140–122). All cells were maintained at 37°C and kept under 5% CO_2_.

#### Mouse model

All experimental procedures were approved by the Institutional Animal Care and Use Committee of Saint Louis University (protocol # 2764). PGRN knockout (KO) (i.e., *Grn*^−/−^) mice were generated as described,^15^ maintained on a C57BL/6J background (backcrossed >8 generations) and genotyped by real-time PCR (Transnetyx). Mice were housed in a pathogen-free barrier facility with a 12-h light/12-h dark cycle and allowed food (standard chow) and water *ad libitum*. Experimental cohorts (*Grn*^*+/−*^; *Grn*^−/−^) were generated by crossing heterozygous mice, and their wild-type (WT) (*Grn*^*+/+*^) littermates were used for comparisons. Relative findings in males and females (used in equal amounts) were comparable, and results were therefore combined. Mouse tissues were examined at 2–4 months of age when brains of PGRN knockout mice reportedly have impairments in autophagy and lysosomal functions mainly in PGRN-rich microglial cells, as opposed to the neuronal aberrations that are more prominently seen months later.^122^

### Method details

#### Materials

The following were used: aPKC inhibitor, 2-acetyl-cyclopentane-1,3-diketone (ACPD) (Sigma, R426911) (characterized in Sajan et al.^82^^,^^86^^,^^87^); human recombinant PGRN (R&D Systems, 2420-PG-50); JNK inhibitor JNK-IN-7 (Cayman Chemicals, 28831);^80^^,^^81^ and Insulin (Sigma, I9278).

#### Generation of PGRN knockout HMC3 cells

KO cells were generated by CRISPR/Cas9 using the following gRNAs: CCCTGAGACGGTAAAGATGCNGG and CCTGCATCTTTACCGTCTCANGG. Single clones were isolated and disruption of the PGRN (*GRN*) allele was confirmed by NGS-based sequencing, qPCR and Western blot analyses (see Figure S1).

#### Cell incubations

As in previous studies,^32^^,^^33^^,^^34^ LA1-5s and HMC3 cells were incubated for 24 h ± vehicle (i.e., controls) or 200 nM insulin, a concentration chosen to ensure maximal activation of both the insulin receptor and the IGF-1 receptor,^37^ and therefore both IRS-1 and IRS-2, and thus both Akt and PKC-λ/ι and other downstream effectors, in particular, NFκB-dependent proinflammatory cytokines (see Figure S5 showing insulin-dependent increases in TNF-α and IL-6). In this regard, note: (a) IGF-1 receptor levels are upregulated in transgenic AD mice^106^; (b) insulin and IGF-1 activate both the insulin receptor and the IGF-1 receptor and maximal effects in brain are seen at 10-100 nM^37^; (c) during its action, insulin is internalized and undergoes intracellular proteolysis, and medium insulin concentrations diminish substantially during 24-h incubations of cultured neural cells (unpublished observations, M.P. Sajan and R.V. Farese); and (d) the hyperinsulinemia seen in high-fat-fed MetS/T2DM mice apparently acts excessively in brain via “spare” insulin receptors and/or IGF-1 receptors to maximally activate both Akt and aPKC^32^^,^^33^^,^^34^; thus, the presently used incubation conditions provided a strong test of the ability of PGRN to block all stimulatory effects of insulin and IGF-1 that, we posit, promote FTD and AD development. Also note that we used the phosphorylation of tyr-612-IRS-1 and tyr-978-IRS-2 as indicators of IRS-1 and IRS-2 activation, and these activations were corroborated by concomitant induction of strong/maximal increases in phosphorylation/activity of downstream kinases, PKC-λ/ι and Akt. In this regard, note that insulin itself, by increasing activities of Akt, aPKC, other PKCs, MAPK, JNK, mTOR and p70 S6K, can simultaneously increase the phosphorylation of various serine residues of IRS-1, including, S302, S307, S318, S629 and S636/639^75^; thus, it cannot be assumed that insulin action is impaired from simple increases in the phosphorylation of these serine residues; indeed, depending upon the context of activation, some of these “inhibitory” serine phosphorylations are required for certain actions of insulin.^128^^,^^129^

#### Cell and tissue preparations and extractions

As described,^32^^,^^33^^,^^34^^,^^86^ cultured cells and mouse brain cortex and other tissues were harvested, flash-frozen in liquid N_2_ and stored at −80°C, and subsequently homogenized in buffer containing 0.25M sucrose, 20 mM Tris/HCl (pH, 7.5), 2 mM EGTA, 2 mM EDTA, 1 mM phenylmethylsulfonyl-fluoride, 20 μg/mL leupeptin, 10 μg/mL aprotinin, 2 mM Na_4_P_2_O_7_, 2 mM Na_3_VO_4_, 2 mM NaF, and 1 μM microcystin, and then supplemented with 1% Triton X-100, 0.6% Nonidet and 150 mM NaCl. Protein concentrations were determined using a Bio-Rad DC Protein Assay Kit II, and samples used for Western and ELISA analyses were stored in Laemmli buffer.

#### Western and ELISA analyses

All comparisons were from samples obtained from the same experiment and analyzed together. The following proteins were analyzed by Western blot using antibodies purchased from sources (indicated in parentheses) that target: (a) enzyme-altering phosphorylations of serine (ser or S), threonine (thr or T) and/or tyrosine (tyr or Y) residues of *p*-tyr-612-IRS-1 (Cell Signaling, 2386), *p*-tyr-978-IRS-2 (MyBioSource, 9211379), *p*-ser-473-Akt (Cell Signaling, 9271), *p*-thr-555-PKC-λ/ι (Invitrogen/Thermofisher, 44-968-G), *p*-ser-176/180-IKKα/β (Cell Signaling, 2697), *p*-ser-529/536-NFκB/p65RelA subunit (Cell Signaling, 3033), *p*-ser-2448-mTOR (Cell Signaling, 2971), *p*-thr-183/p-tyr-185-JNK (Cell Signaling, 9251), *p*-ser-302-IRS-1 (Cell Signaling, 2384), *p*-ser-307-IRS-1 (Cell Signaling, 2381), *p*-ser-616-IRS-1 (Invitrogen/ThermoFisher, 44–550) and *p*-ser-636/639-IRS-1 (Cell Signaling, 2388) (this antibody recognizes both 636 and 639 phosphoserine residues in IRS-1); and (b) total levels of human progranulin (generated as described in Nguyen et al.^127^), mouse progranulin (R&D Systems, AF2557), β-Actin (Cell Signaling, 8457), Vinculin (Cell Signaling, 13901), JNK (Cell Signaling, 9252), IRS-1 (Cell Signaling, 2382), IRS-2 (Cell Signaling, 4502), Akt (Cell Signaling, 9272), PKC-λ/ι (Santa Cruz, sc-376344), IKK (Cell Signaling, 8943), NF-κB/p65RelA (Cell Signaling, 8242), mTOR (Cell Signaling, 2972), BACE1 (Invitrogen/ThermoFisher, PA1-757) and IR-β (Cell Signaling, 3025). TNF-α protein levels were measured by ELISA (kit from Invitrogen/ThermoFisher). Data S1 shows original raw blots of proteins measured and their apparent molecular weights, i.e., kDa, as determined by comparison to molecular weight standards, for Figures 1, 2, 3, 4, 5, 6, and S1–S4.

It may be noted that increases in the phosphorylation of the key tyrosine, serine or threonine amino acids of various signaling proteins were presently considered as reflective of activation only when there was clear evidence that the activity or level of downstream factors had been significantly altered, as follows: PGRN-dependent phosphorylation of JNK led to increases in phosphorylation of ser-302-IRS-1 and ser-307-IRS-1; JNK-dependent phosphorylation of ser-302-IRS-1 and ser-307-IRS-1 led to inhibition of IRS-1 activation; insulin receptor-dependent phosphorylation of tyr-612-IRS1 and/or tyr-978-IRS-2 led to activation of PKC-λ/ι and/or Akt; phosphorylation of thr-555-PKC-λ/ι led to activation of IKKα/β and/or NF-κB; and phosphorylation of ser-529/536-NFκB led to increased expression and/or levels of TNF-α, IL-1β and IL-6. Further note that we had previously shown that changes in the phosphorylation status of both thr-555-PKC-λ/ι and ser-473-Akt, as induced by insulin or altered by obesity/diabetes and aPKC and Akt inhibitors, are paralleled by comparable relative changes in actual enzyme activity of aPKC and Akt, as measured by specific immunoprecipitation of aPKC or Akt followed by^32^ PO_4_-labeling [i.e., transfer of^32^ (PO_4_) from γ-labelled ATP] of a specific substrate during the incubation of these immunoprecipitates (see parallel comparisons of both methods in the same samples in Sajan et al.^86^).

#### mRNA analyses

Gene expression was analyzed by real-time quantitative PCR (qPCR) of total RNA isolated from flash frozen tissues with the RNeasy Mini kit (Qiagen, 74106) using on-column DNase digestion (Qiagen, 79256). RNA was reverse transcribed to obtain cDNA using the iScript cDNA synthesis kit (Bio-Rad, 1708891), and qPCR was performed using PowerUp SYBR Green Master Mix (ThermoFisher, A25777) with a Bio-Rad CFX384 Real-Time System. Primers sequences are provided in Table S1. Results for qPCR were normalized to the housekeeping gene *CYCLO* and analyzed by the comparative C_T_ method.

### Quantification and statistical analysis

Data in this study are presented as mean ± SEM. Comparisons among multiple groups were made using one-way analysis of variance (ANOVA) with Tukey post hoc test, and differences with *p* values less than 0.05 were considered statistically significant.
