# Supplementary material for: Progranulin deficiency in the brain activates an insulin signaling pathway that may promote neurodegeneration
Source: iScience. 2026 Apr 12;29(5):115720. doi: 10.1016/j.isci.2026.115720 (PMC13141651; doi:10.1016/j.isci.2026.115720)
Supplement: Document S1. Figures S1–S5, Table S1, and Data S1 [file mmc1.pdf]

iScience, Volume *29*

Supplemental information

Progranulin deficiency in the brain activates an insulin signaling pathway that may promote neurodegeneration

Mini P. Sajan, Geetika Aggarwal, Joel Jihwan Hwang, Denise M. Smith, Denise R. Cooper, Mildred Acevedo Duncan, Barbara C. Hansen, Andrew D. Nguyen, and Robert V. Farese

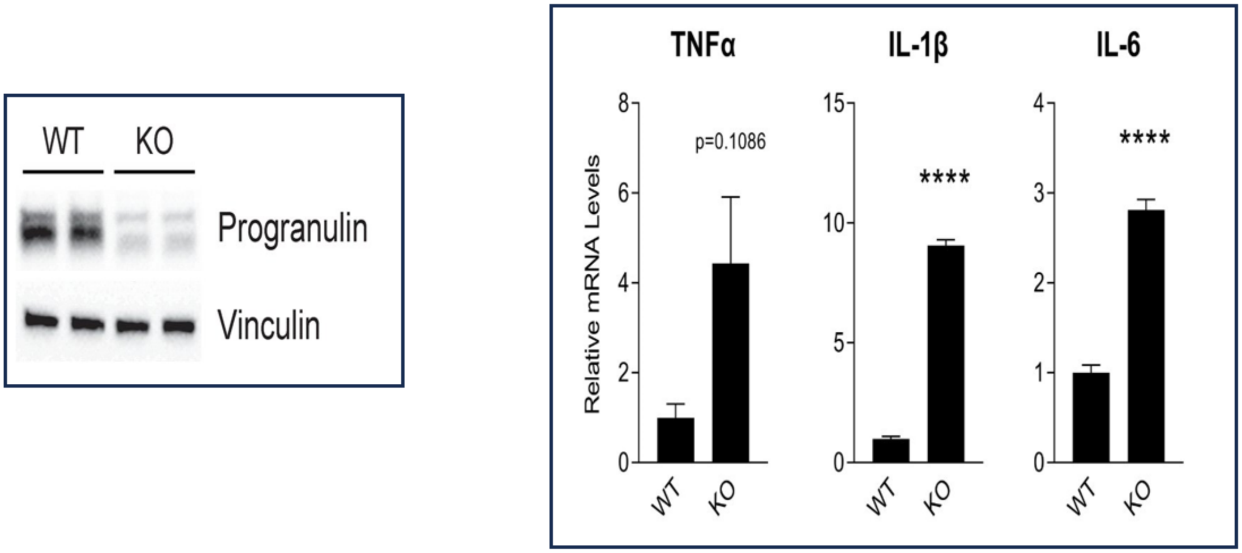

Figure S1. CRISPR/Cas9 knockout (KO) of Progranulin (PGRN) (left panel) increases mRNA levels (right panel) of tumor necrosis factor-alpha (TNF-α), Interleukin-1beta (IL-1β) and Interleukin-6 (IL-6) in HMC3 microglial cells. Asterisks, ****, indicate P<0.0001 (N=4) (ANOVA).

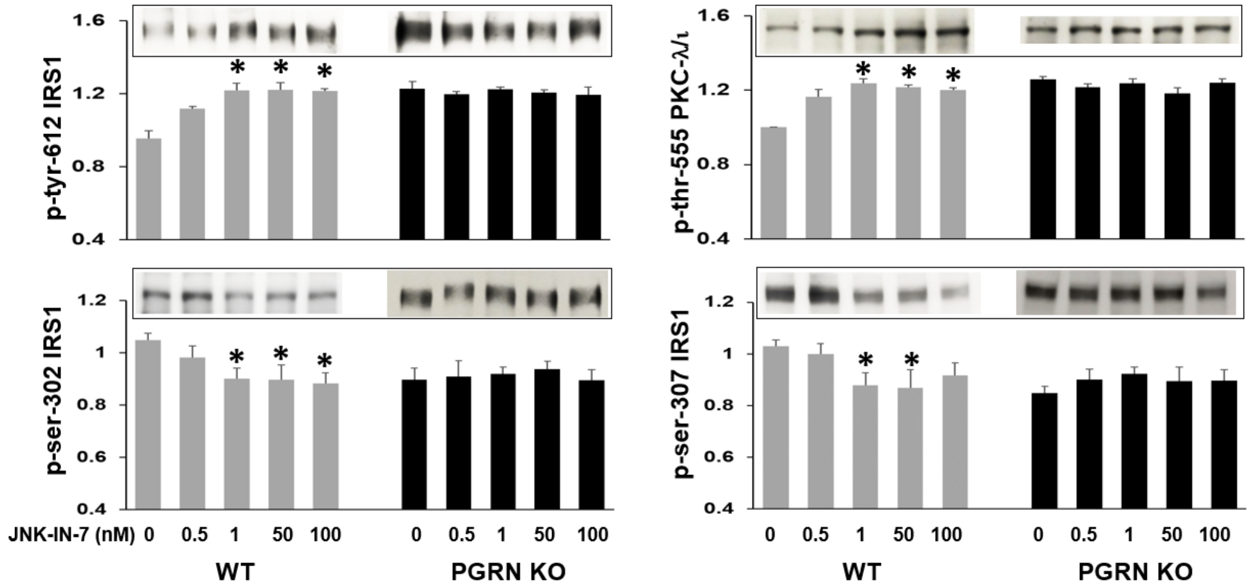

Figure S2. Failure of JNK inhibitor, JNK-IN-7, to alter activation of IRS-1 and activities of PKC-λ/ι and NFκB in PGRN-deficient HMC3 microglial cells. Cells were incubated for 24 hours with indicated concentrations of JNK-IN-7, as in Figure 5. Values in bargrams are Mean ± SEM of 3-4 comparisons of findings in JNK-IN-7-treated cells relative to findings in untreated control cells, with the latter set as unity. Asterisks (*) indicate a significant difference of $P<0.05$ (ANOVA) between treated versus untreated control samples.

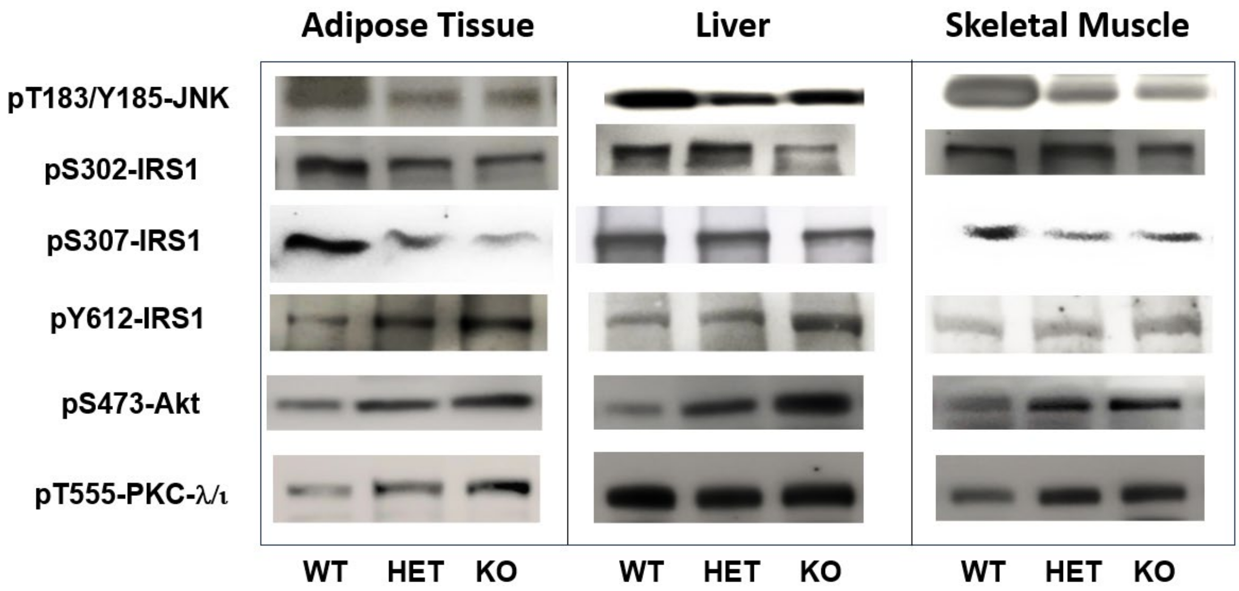

Figure S3. Heterozygous (HET) and homozygous knockout (KO) of progranulin (PGRN) in mice (a) diminishes activity of JNK and phosphorylation of ser-302-IRS-1 and ser-307-IRS-1 in adipose tissue, liver and skeletal muscle, and (b) concomitantly increases activity of both Akt and PKC-λ/ι in adipose tissue and skeletal muscle, and activity of Akt, but not PKC-λ/ι, in liver.

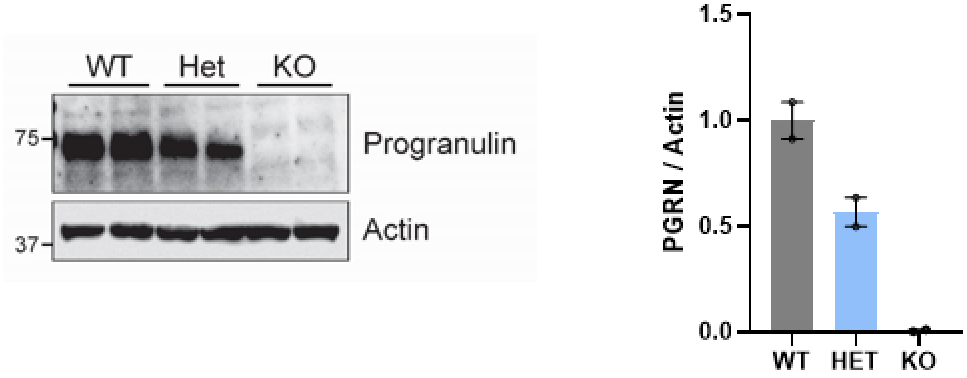

Figure S4. Western blot showing graded decreases in PGRN levels in mouse brain cortex in heterozygous (Het) and homozygous (KO) knockout mice.

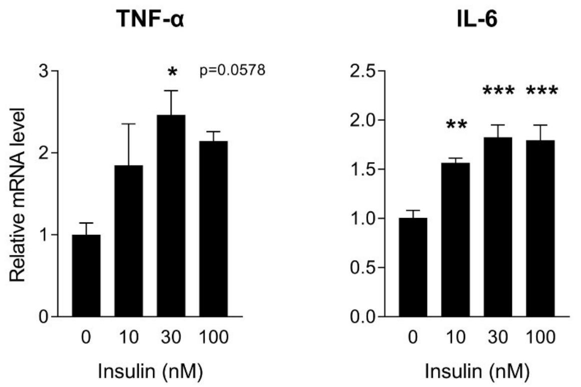

Figure S5. Insulin increases mRNA levels of TNF-α and IL-6 in HMC3 microglial cells. Cells were incubated for 3 hours with indicated concentrations of insulin. Values are Mean ± SEM of 3-4 determinations. Asterisks indicate: *, $P<0.05$; **, $P<0.01$; ***, $P<0.001$ (ANOVA).

Table S1. qPCR primer sequences.

Gene	Forward Primer	Reverse Primer
CYCLO	GGAGATGGCACAGGAGGAAA	CCGTAGTGCTTCAGTTTGAAGTTCT
GRN	AGGAGAACGCTACCACGGA	GGCAGCAGGTATAGCCATCTG
TNF-α	GCTCCAGGCGGTGCTTGTTC	TGGGGAACTCTTCCCTCTGGGG
IL-1β	GCAGGCCGCGTCAGTTGTTG	TCTGTGGGCAGGGAACCAGC
IL-6	TCCACAAGCGCCTTCGGTCC	GGGGGTACTGGGGCAGGGAA

RAW Images-Fig 1.

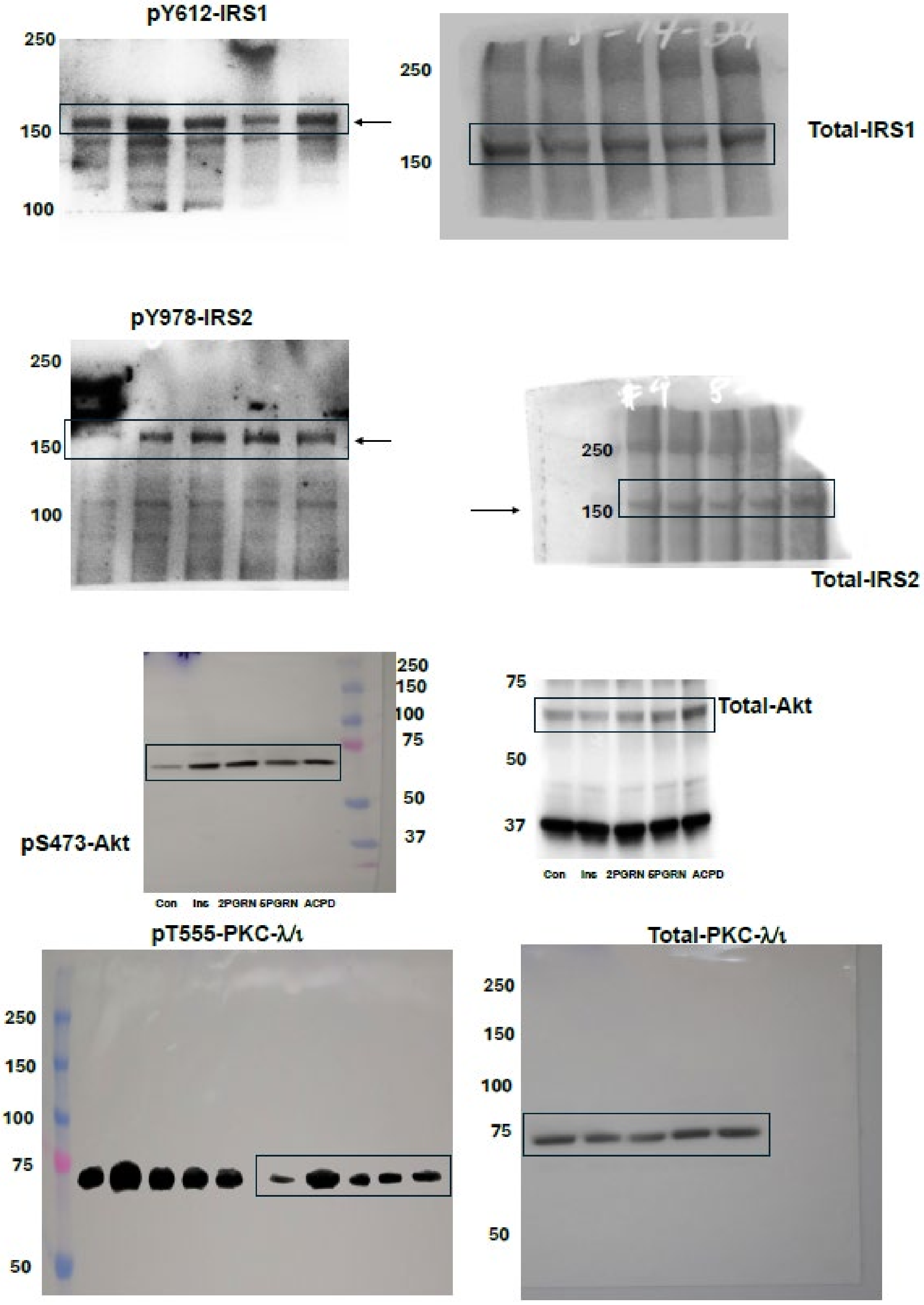

Data S1. Original western blot images. Original images of proteins analyzed in Figures 1-6 and Figures S1-S4, with apparent molecular weights. Note that, after protein transfer from gels to membranes, membranes were cut to enable blotting of several proteins from each membrane. Images are continued on the following pages.

RAW Images-Fig 1.

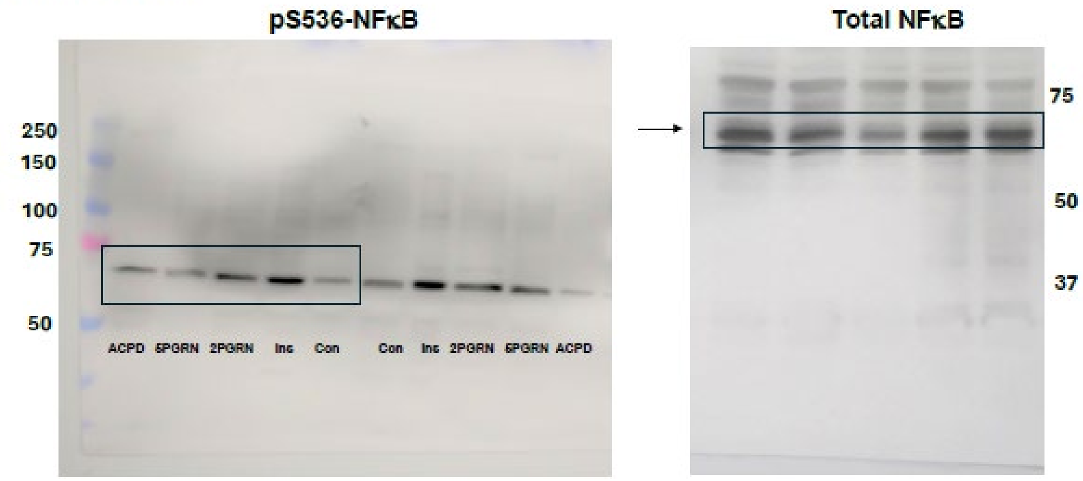

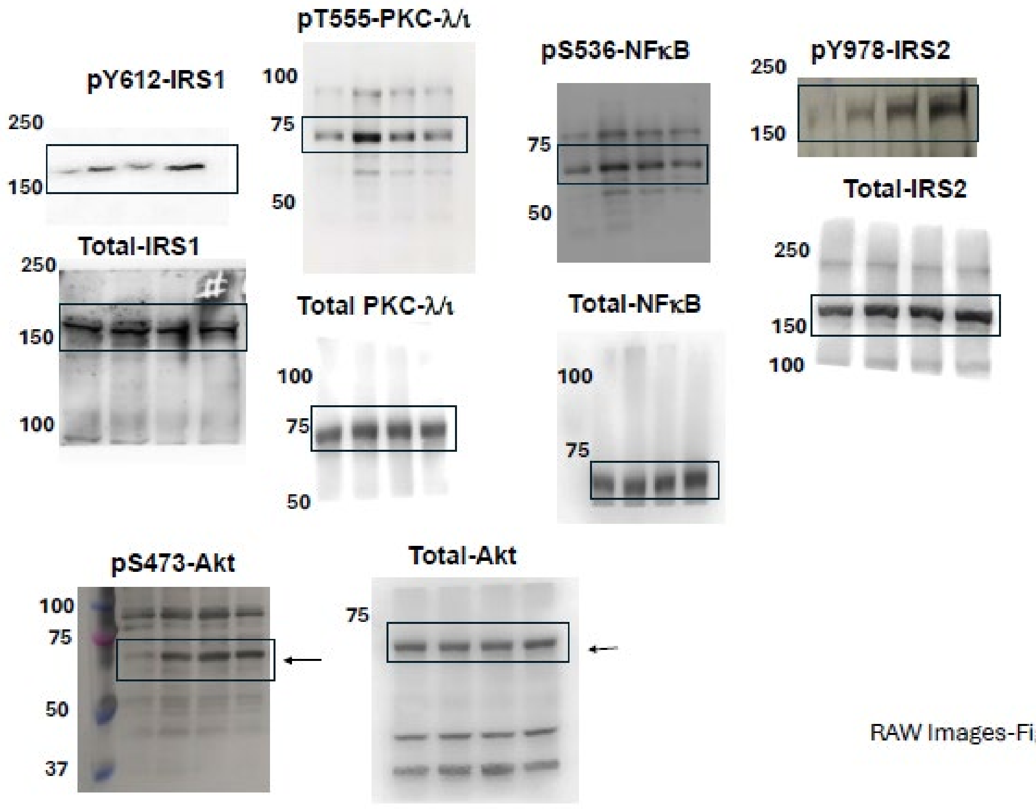

RAW Images-Fig 2.

RAW Images-Fig 3.

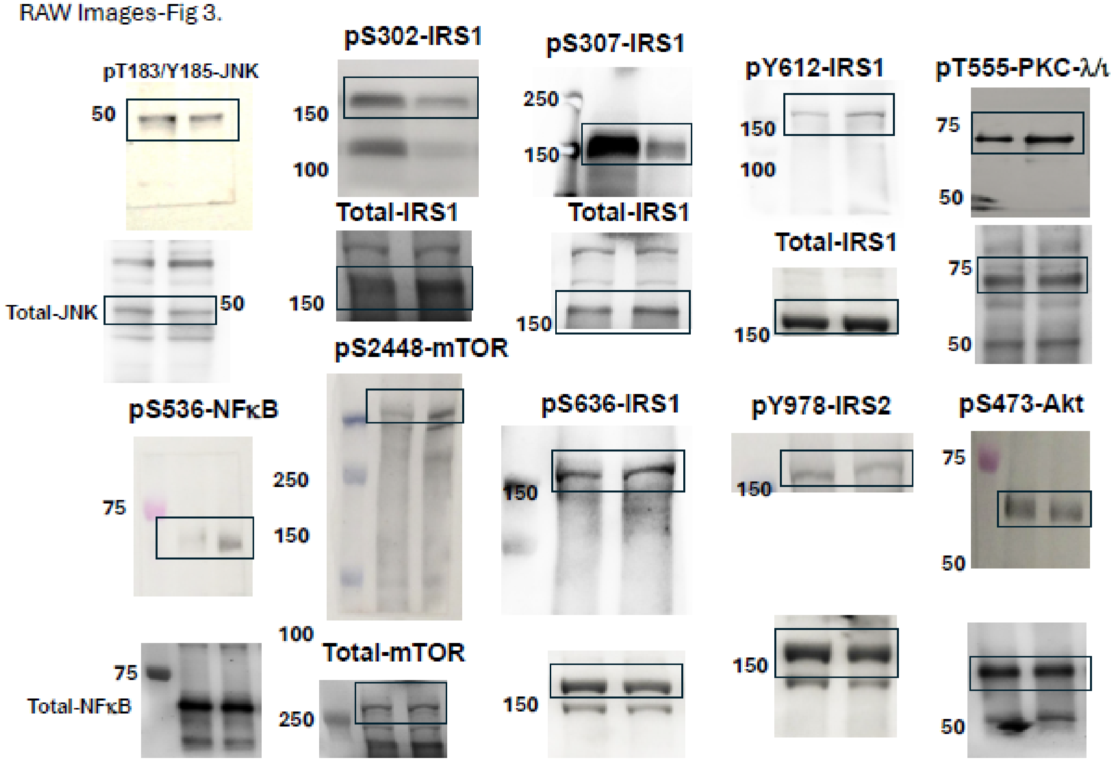

RAW Images-Fig 4.

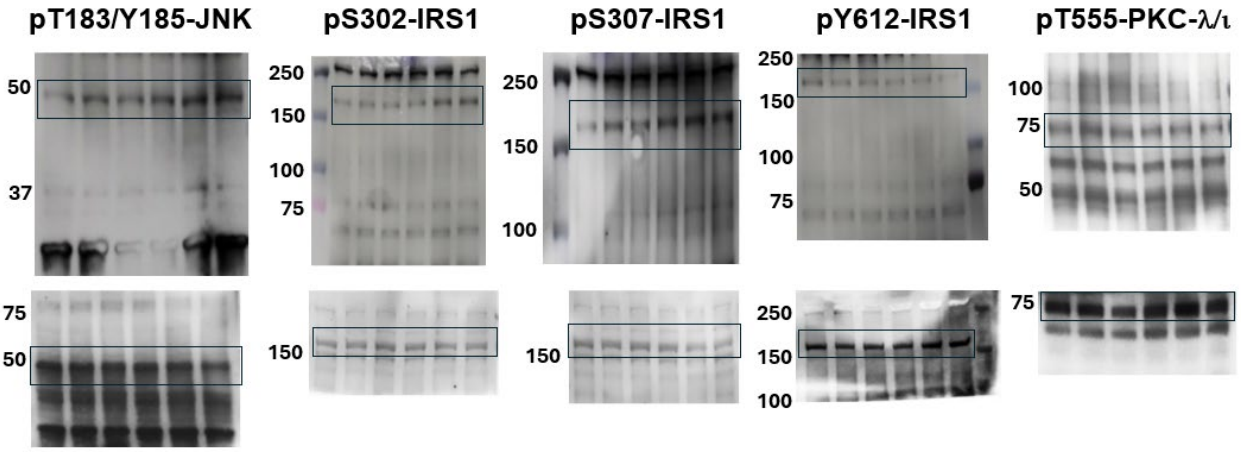

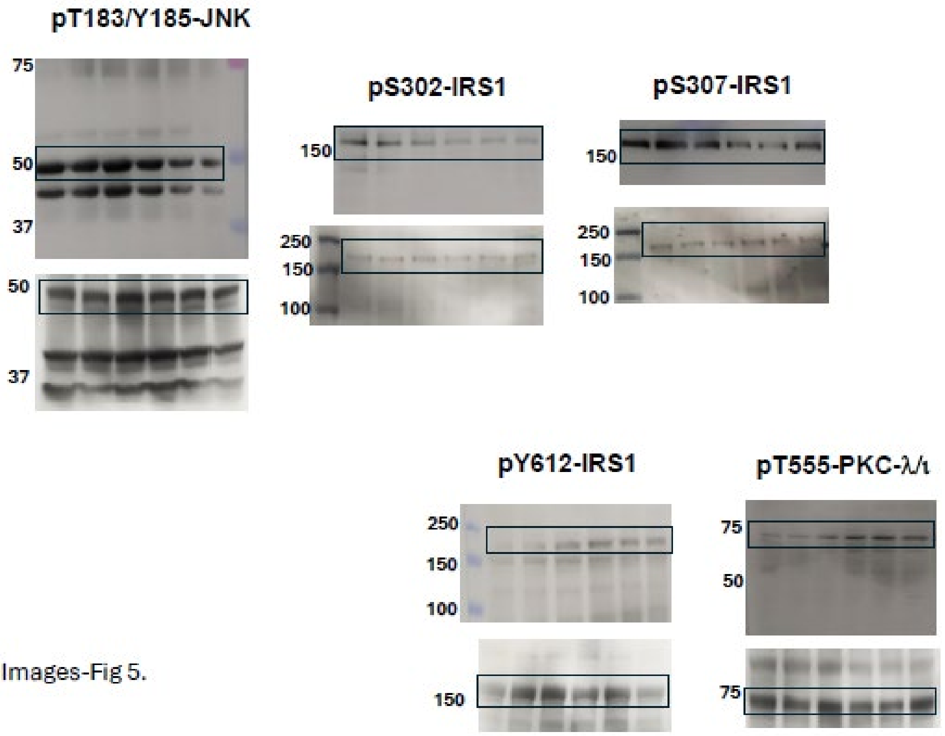

RAW Images-Fig 5.

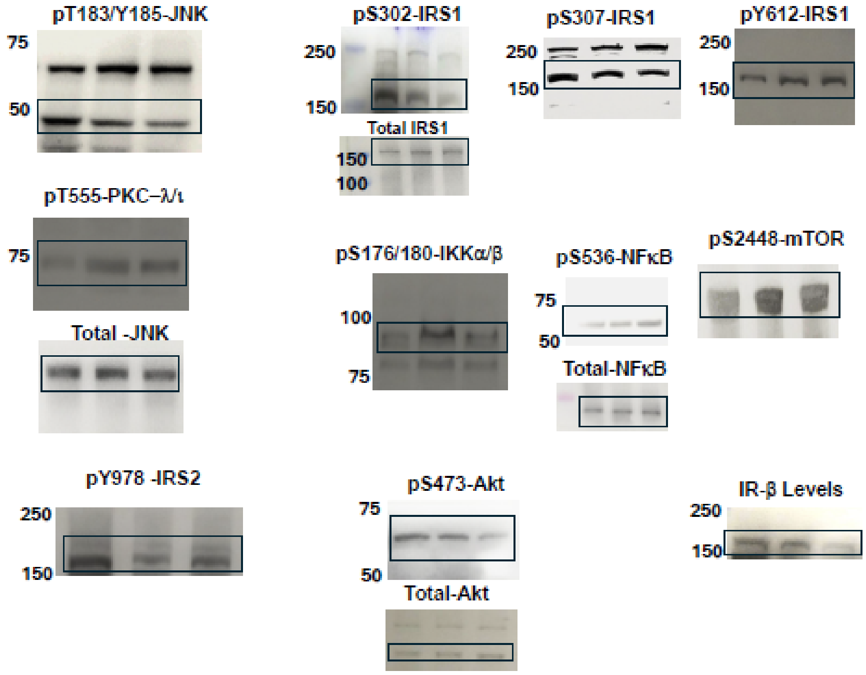

RAW Images-Fig 6.

RAW Images-Fig S1.

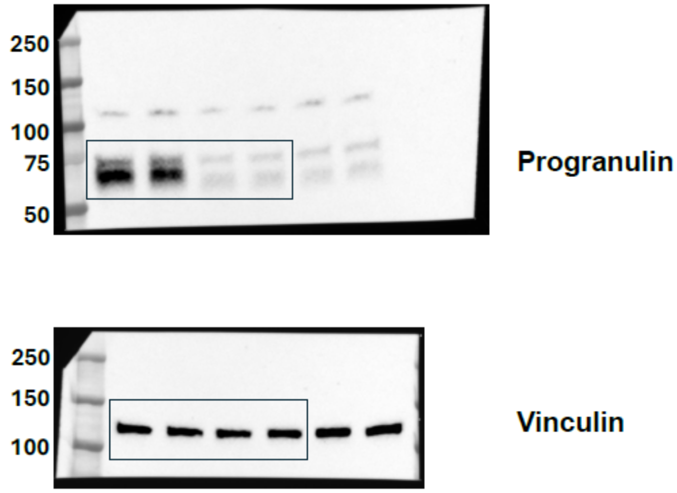

RAW Images-Fig S2.

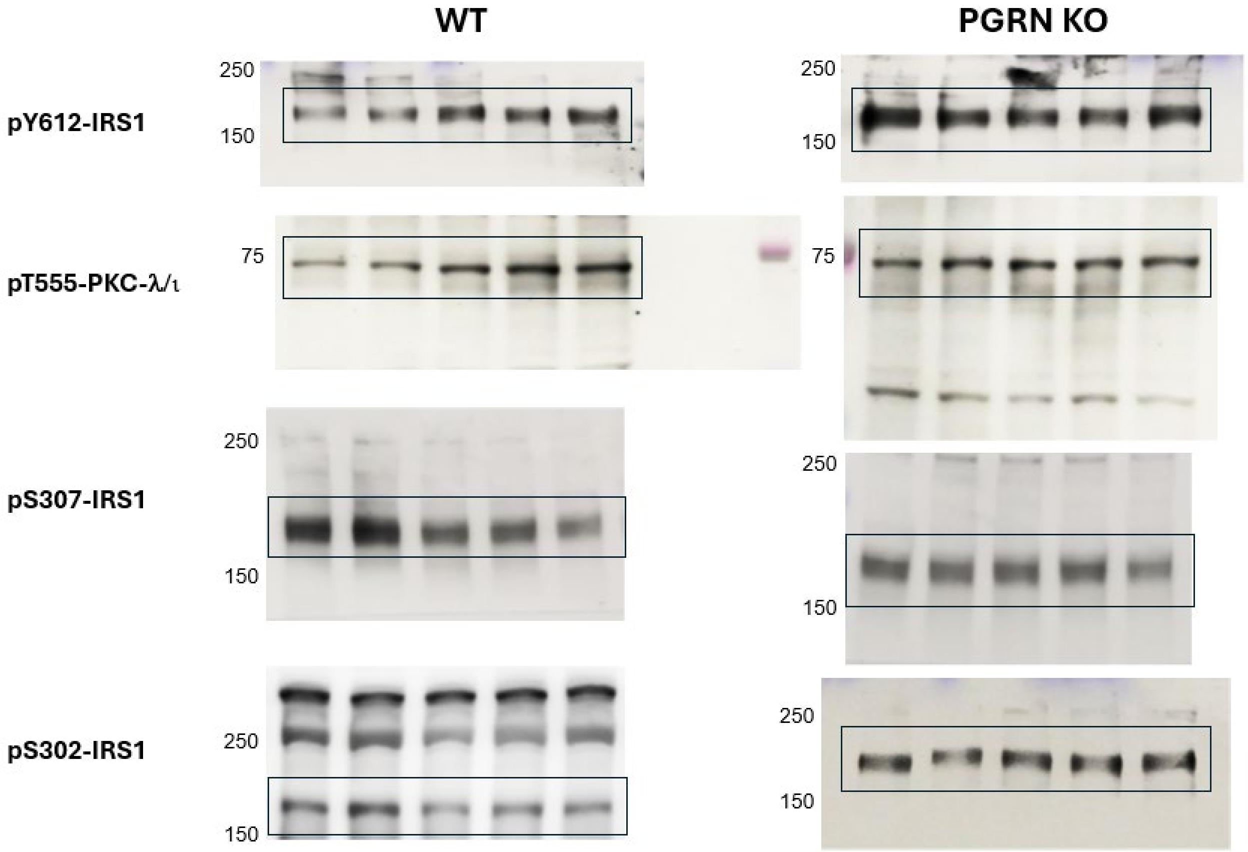

RAW Images-Fig S3

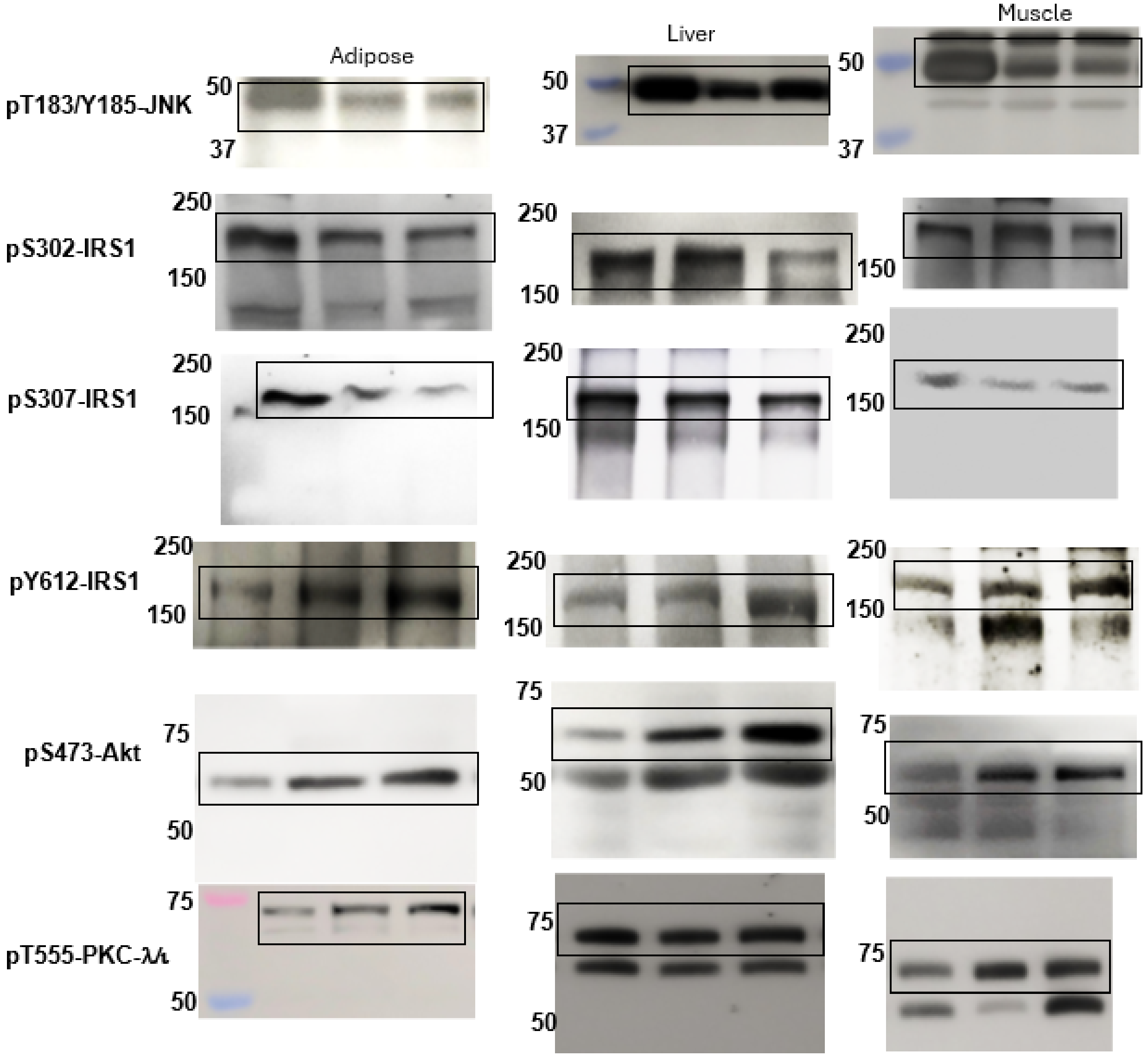

RAW Images-Fig S4.

250
150
100
75
50

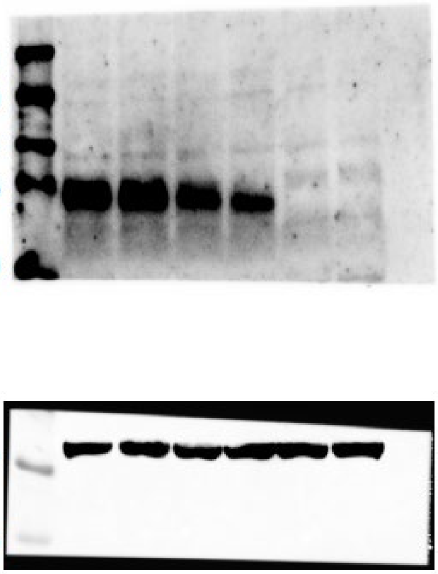

Progranulin

37
25

Actin
